# Supplementary material for: MatchMiner: an open-source platform for cancer precision medicine
Source: NPJ Precis Oncol. 2022 Oct 6;6:69. doi: 10.1038/s41698-022-00312-5 (PMC9537311; doi:10.1038/s41698-022-00312-5)
Supplement: Supplementary file 4 — REPORTING SUMMARY [file 41698_2022_312_MOESM4_ESM.pdf]

nature portfolio

Corresponding author(s): Harry Klein and Tali Mazor

Last updated by author(s): August 11, 2022

Reporting Summary

Nature Portfolio wishes to improve the reproducibility of the work that we publish. This form provides structure for consistency and transparency in reporting. For further information on Nature Portfolio policies, see our Editorial Policies and the Editorial Policy Checklist.

Statistics

For all statistical analyses, confirm that the following items are present in the figure legend, table legend, main text, or Methods section.

n/a	Confirmed	
☐	☒	The exact sample size (*n*) for each experimental group/condition, given as a discrete number and unit of measurement
☒	☐	A statement on whether measurements were taken from distinct samples or whether the same sample was measured repeatedly
☐	☒	The statistical test(s) used AND whether they are one- or two-sided<br>*Only common tests should be described solely by name; describe more complex techniques in the Methods section.*
☒	☐	A description of all covariates tested
☒	☐	A description of any assumptions or corrections, such as tests of normality and adjustment for multiple comparisons
☒	☐	A full description of the statistical parameters including central tendency (e.g. means) or other basic estimates (e.g. regression coefficient) AND variation (e.g. standard deviation) or associated estimates of uncertainty (e.g. confidence intervals)
☒	☐	For null hypothesis testing, the test statistic (e.g. *F*, *t*, *r*) with confidence intervals, effect sizes, degrees of freedom and *P* value noted<br>*Give P values as exact values whenever suitable.*
☒	☐	For Bayesian analysis, information on the choice of priors and Markov chain Monte Carlo settings
☒	☐	For hierarchical and complex designs, identification of the appropriate level for tests and full reporting of outcomes
☒	☐	Estimates of effect sizes (e.g. Cohen's *d*, Pearson's *r*), indicating how they were calculated

Our web collection on statistics for biologists contains articles on many of the points above.

Software and code

Policy information about availability of computer code

Data collection	For the DFCI trial analysis, extraction of genes and cancer types was performed by recursively traversing trial CTMLs with custom scripts and summarized with R ggplot2 and gtsummary.<br><br>For the impact analysis, consent dates were extracted from DFCI's OnCore trial registration database. For our analytic cohort, time from MatchMiner ingestion date to consent date was calculated on a DFCI HIPAA compliant server.
Data analysis	For the DFCI trial analysis, all cancer types from trials were annotated with their corresponding OncoTree metatype (OncoTree version: oncotree_legacy_1.1). Trial phases and disease center were extracted from the summary field of trial json files with Python (v. 2.7) pandas json_normalize function.<br><br>For the impact analysis, filtering was performed with Python (v. 2.7) pandas commands and the MatchMiner ingestion date to consent date time period was analyzed for MMC and non-MMC using a two-tailed Wilcoxon rank sum test with R gtsummary.

For manuscripts utilizing custom algorithms or software that are central to the research but not yet described in published literature, software must be made available to editors and reviewers. We strongly encourage code deposition in a community repository (e.g. GitHub). See the Nature Portfolio guidelines for submitting code & software for further information.

Data

Policy information about availability of data

All manuscripts must include a data availability statement. This statement should provide the following information, where applicable:

- Accession codes, unique identifiers, or web links for publicly available datasets
- A description of any restrictions on data availability
- For clinical datasets or third party data, please ensure that the statement adheres to our policy

The time intervals between MatchMiner ingestion date and consent date for each patient (which are presented in the Results section "Impact of MatchMiner on PM trial consent" and in Figure 4) are available in Supplementary Tables 3 and 4. Detailed trial eligibility data (which are discussed in results section "Features of PM trials in MatchMiner" and in Figure 3) are unavailable due to contractual restrictions with clinical trial sponsors regarding the sharing of potentially proprietary eligibility criteria.

Human research participants

Policy information about studies involving human research participants and Sex and Gender in Research.

Reporting on sex and gender	We report the gender of MatchMiner consents in the impact analysis result. We obtained consent for their use in our manuscript. We had 106 female participants and 53 male participants. Reporting gender was used to provide context for our patient population.
Population characteristics	From our impact analysis, the average age of MMC patients is 60 years old (min=8, max=86) with most patients between 50-64 years old (n=70, 44%) (Supplementary Table 2). 67% of patients are female (n=106) and 33% are male (n=53). Most patients are white (n=137, 86%), followed by African American (n=7, 4%), Asian (n=6, 4%), and other/unknown (n=6, 4%).
Recruitment	This was a retrospective study. No subjects were recruited.
Ethics oversight	This study was approved by the institutional investigational review board at Dana-Farber Cancer Institute, which determined that neither the physicians nor the patients needed to be consented for this retrospective study.

Note that full information on the approval of the study protocol must also be provided in the manuscript.

Field-specific reporting

Please select the one below that is the best fit for your research. If you are not sure, read the appropriate sections before making your selection.

☒ Life sciences ☐ Behavioural & social sciences ☐ Ecological, evolutionary & environmental sciences

For a reference copy of the document with all sections, see nature.com/documents/nr-reporting-summary-flat.pdf

Life sciences study design

All studies must disclose on these points even when the disclosure is negative.

Sample size	For the impact analysis, sample size for the analytic cohort was determined based on the number of MatchMiner consents and the number of consents on those same PM trials.
Data exclusions	No data was excluded from the impact trial analysis.
Replication	Because our impact analysis was a retrospective study over an approximately 4 year period with many institution specific variables, we do not think our study is repeatable.
Randomization	Because we conducted a retrospective study for the impact analysis, no randomization occurred.
Blinding	Blinding was not relevant for our impact analysis, because it was a retrospective measure.

Reporting for specific materials, systems and methods

We require information from authors about some types of materials, experimental systems and methods used in many studies. Here, indicate whether each material, system or method listed is relevant to your study. If you are not sure if a list item applies to your research, read the appropriate section before selecting a response.

Materials & experimental systems

n/a	Involved in the study
☒	☐ Antibodies
☒	☐ Eukaryotic cell lines
☒	☐ Palaeontology and archaeology
☒	☐ Animals and other organisms
☒	☐ Clinical data
☒	☐ Dual use research of concern

Methods

n/a	Involved in the study
☒	☐ ChIP-seq
☒	☐ Flow cytometry
☒	☐ MRI-based neuroimaging
